# Revealing Phenotypic Differentiation in *Ochetobius elongatus* from the Middle Yangtze River Through Geometric Morphometrics

**DOI:** 10.3390/ani15192870

**Published:** 2025-09-30

**Authors:** Fangtao Cai, Zhiyuan Qi, Ziheng Hu, Dongdong Zhai, Yuanyuan Chen, Fei Xiong, Hongyan Liu

**Affiliations:** 1 School of Life Sciences, Hubei Engineering Research Center for Protection and Utilization of Special Biological Resources in the Hanjiang River Basin, Jianghan University, Wuhan 430056, China; 18311558834@163.com (F.C.); q15055974063@163.com (Z.Q.); hzh01f@163.com (Z.H.); zhaidongdong@jhun.edu.cn (D.Z.); yychen@jhun.edu.cn (Y.C.); xiongfei@jhun.edu.cn (F.X.); 2 Hubei Key Laboratory of Environmental and Health Effects of Persistent Toxic Substances, Jianghan University, Wuhan 430056, China

**Keywords:** *Ochetobius elongatus*, geometric morphometrics, phenotypic plasticity, phenotypic differentiation, the endangered species

## Abstract

**Simple Summary:**

Phenotypic differentiation reflected species adaptation to different habitats. *Ochetobius elongatus* was once an economically important species in the south of the Yangtze River, China. However, due to environmental degradation and human activities, its wild populations have declined drastically, and it is now classified as a critically endangered (CR) species. Following the implementation of the Ten-Year Fishing Ban in the Yangtze River, *O. elongatus* has reappeared in certain sections of the mainstem, the tributary and the river-connected lakes of the middle Yangtze River, which could provide valuable samples for studying its morphological variation and habitat adaptation. Using geometric morphometrics based on 14 landmarks, this study revealed significant phenotypic differentiation among *O. elongatus* populations from distinct habitats. These results indicated that phenotypic plasticity might underlie their adaptation to diverse aquatic environments. The findings provided important insights into the survival strategies of this species and could support its conservation and restoration efforts.

**Abstract:**

*Ochetobius elongatus*, a critically endangered (CR) fish species of the Yangtze River Basin in China, has experienced a severe decline in its wild population. Understanding its mechanisms of phenotypic variation is essential for developing effective conservation and restoration strategies. Using geometric morphometrics based on 14 landmarks, we examined the phenotypic difference among five populations from the mainstem, the tributary, and the river-connected lakes of the middle Yangtze River. The results showed that significant phenotypic divergence was detected between river and lake populations. River individuals exhibited a more elongated body, smaller head, inferior mouth position, larger operculum, and narrower caudal peduncle, whereas lake individuals showed a deeper body, and anterior shift in the origin of pelvic fin. The first canonical variable effectively distinguished river and lake populations, with the accuracy of both original and cross-validation classification exceeding 90%, indicating that habitat heterogeneity was the primary driver of phenotypic differentiation. No significant correlation was found between morphological distance and geographical distance. Water temperature, flow velocity, water depth, and food abundance significantly influenced phenotypic variation, but their individual effects were limited, which suggested that environmental shaping of morphology depended more on synergistic effects. Our findings provide important insights into the adaptive evolution of this critically endangered species and offer a scientific basis for conservation efforts.

## 1. Introduction

Intraspecific phenotypic variation, often shaped by selective pressures from different habitats, is a key strategy for species to adapt to heterogeneous environment [1,2,3]. As the most morphologically diverse group of vertebrates, fish are widely distributed across diverse aquatic habitats, making them ideal models for understanding the mechanisms by which habitat variation causes phenotypic differences [4,5]. Fish were highly sensitive to environmental changes, and their morphology could be influenced by factors such as water temperature, flow velocity, and food abundance [6,7]. Phenotypic plasticity enabled fish to respond rapidly to different habitats, resulting in obviously adaptive morphological changes [8,9]. For instance, flow gradients could directly induce phenotypic plasticity in species such as *Curimata mivartii* and *Pimelodus grosskopfii*, which modified their body shape to cope with varying water velocity [10]. Variations in food resources could drive adaptive phenotypic divergence in *Salvelinus alpinus*, with planktivorous populations exhibiting a deeper body shape, while benthivorous populations showed a slender body and larger head [11]. Habitat pressure influenced body structure, as seen in *Caquetaia kraussii*, where populations in river sections displayed a slenderer body and a larger operculum compared to those in shallow swamp areas [12].

The middle Yangtze River forms a complex network comprising the mainstem, the tributary, and the river-connected lakes (e.g., Dongting Lake and Poyang Lake), which together create a distinct habitat gradient [13,14]. The mainstem are characterized by high flow velocity and deep channels with limited food resources, the tributary exhibit moderate flow and depth, supporting richer plankton and benthic communities, and the river-connected lakes experience large water-level fluctuations and contain extensive wetland areas with high biodiversity and substantial biomass of food organisms [15,16]. These systematic differences in hydrology and resource availability may exert selective pressures on fish survival, feeding, and growth, potentially driving significant phenotypic adaptive divergence across these habitats.

*O. elongatus* was once an economically important species in the south of the Yangtze River, China [17,18]. However, due to habitat fragmentation, fishing pressure, and environmental pollution, its wild populations have declined drastically over the past four decades [19,20,21]. By the late 20th century, the species has nearly disappeared from field surveys. Now it is classified as a critically endangered (CR) species by both the China Red List of Vertebrates and the China Biodiversity Red List [22,23]. Following the implementation of the Ten-Year Fishing Ban in the Yangtze River in 2021, *O. elongatus* has reappeared in certain sections of the mainstem, the tributary, and the river-connected lakes of the middle Yangtze River, which offers a potential opportunity for population recovery. To develop effective conservation and restoration strategies, it is essential to clarify the population structure and ecological divergence within the species. Zhai et al. [24] found no significant genetic differentiation among populations in the middle Yangtze River using molecular markers, but genetic homogeneity was not synonymous with phenotypic homogeneity [7]. Therefore, examining phenotypic divergence through morphometric approaches can provide a scientific basis for precisely targeted conservation measures.

Geometric morphometrics is an analytical approach that quantifies biological morphological features through mathematical modeling and statistical methods to decipher patterns of phenotypic variation, their underlying causes, and ecological adaptations [5,25]. The methodology encompasses linear measurement, truss network, and geometric morphometrics [26,27]. In our previous study, we used the methods linear measurement and truss network for a preliminary morphological comparison of the populations of *O. elongatus*. However, as the two methods rely on linear variables, the results were highly correlated with individual size of the fish, thus making it difficult to effectively disentangle size-related changes from genuine shape variation. Although allometric adjustments (e.g., isometric standardization) can partially mitigate size effects, the traditional morphometrics remain limited in resolving subtle shape differences [26,28]. Geometric morphometrics use digitally annotated landmarks to accurately capture spatial configurations of biological structures. Generalized Procrustes Analysis (GPA) have effectively eliminated non-shape variations related to size, position, and orientation [29,30]. This enables more direct and intuitive quantification and comparison of morphological variation. Consequently, geometric morphometrics has become a predominant tool for studies of population discrimination and phenotypic divergence in fishes [27,31,32].

This study used geometric morphometrics to analyze phenotypic differences of the populations of *O. elongatus* in the mainstem, the tributary, and the river-connected lakes in the middle Yangtze River. The aims were to clarify the following questions: (1) whether significant phenotypic divergence existed among the populations from different habitats, (2) what were the key anatomical regions of phenotypic differentiation and their potential adaptive implications, and (3) whether the phenotypic variation correlated with geographical distance and environmental factors.

## 2. Materials and Methods

### 2.1. Study Area and Sampling

**Figure 1:**
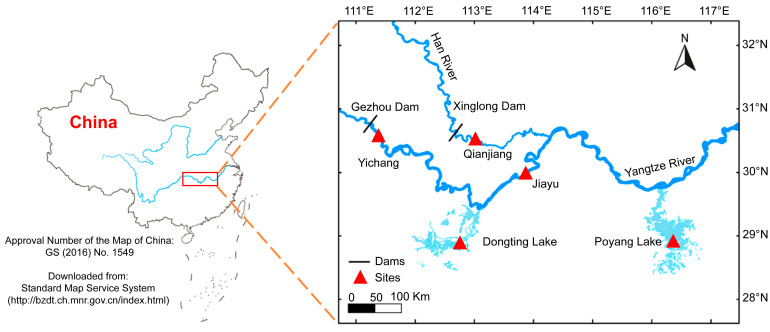
Sampling sites for *O. elongatus* in the middle Yangtze River. Map source: http://bzdt.ch.mnr.gov.cn/index.html, accessed on 20 May 2025.

The study area encompassed three typical habitats in the middle Yangtze River: the mainstem river (Yichang and Jiayu section), the tributary river (Qianjiang section), and the river-connected lakes (Dongting and Poyang Lake) (Figure 1).

Sampling was conducted from 2022 to 2024 across these regions, covering two periods each year: May to July and September to November. In each region, a fish survey was performed continuously for 10 days per period. Over the three-year study, each region accumulated a total of 60 sampling days. All sampling activities were carried out under a special scientific fishing permit and under the supervision of local fisheries authorities, using stationary gillnets of multiple mesh sizes (2, 6, 10, and 14 cm), each of which measured 50 m in length and 2 m in height. Species identification followed the criteria in Fauna Sinica: Osteichthyes, Cypriniformes (Volume II) [17]. Biological measurements were taken immediately after capture. A total of 95 valid specimens were obtained (Table S1), which were frozen and stored for subsequent geometric morphometric analysis.

### 2.2. Data Collecting and Processing

**Figure 2:**
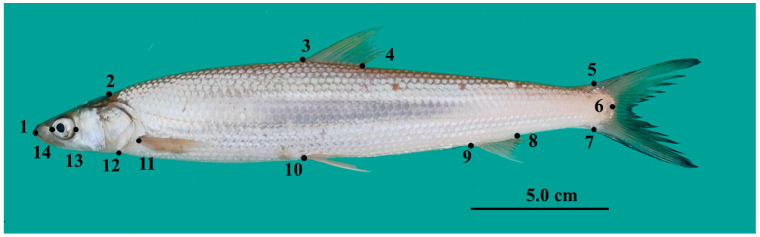
Position of the landmarks used in the geometric morphometric analysis: 1: oral position; 2: distal tip of occiput; 3: origin of dorsal fin; 4: posterior end of dorsal fin base; 5: upper insertion of caudal fin base; 6: distal tip of caudal peduncle; 7: lower insertion of caudal fin base; 8: posterior end of anal fin base; 9: origin of anal fin; 10: origin of pelvic fin; 11: origin of pectoral fin; 12: origin of the preopercle on the ventral; 13: the posterior margin of the eye; and 14: the anterior margin of the eye.

Lateral morphological images of *O. elongatus* were captured vertically using a digital camera, with a fixed height and focal length. Specimens were positioned to ensure natural body extension, full fin expansion, and alignment of the body axis parallel to the image plane. A standardized imaging with a light-colored background was used to minimize postural variation. Specimen placement, camera angle, and focal point were kept consistent across sessions. A scale bar (accuracy: 1 mm) was included for spatial calibration to ensure reproducibility and coordinate consistency. Images were converted to TPS format using tpsUtil (v1.83) software. A total of 14 landmarks were then digitized on each image using tpsDig2 (v2.32) software (Figure 2).

To assess data reliability, 20% of the samples were randomly selected and re-digitized by the same operator after an interval of at least two weeks. Procrustes ANOVA was performed to evaluate sources of variation, and the significance of operator-induced error (*p*-value) was assessed along with the intraclass correlation coefficient (ICC) to ensure measurement repeatability [33,34].

### 2.3. Geometric Morphometric Analysis

Generalized Procrustes Analysis (GPA) was performed in MorphoJ (v1.08) to align all specimens by removing differences in position, scale, and orientation, yielding a consensus configuration representing the overall morphology and group-specific mean shapes. Thin-plate spline (TPS) deformation grids were generated to visualize shape changes in key anatomical regions between populations. Principal component analysis (PCA) was conducted based on the Procrustes coordinates. Principal components (PCs) accounting for over 95% of cumulative variance were retained. Canonical variate analysis (CVA) was utilized to quantify phenotypic differences among the populations, with Mahalanobis and Procrustes distances used as metrics. The Mahalanobis distance, derived from the covariance matrix, measured the degree of morphological overlap among groups by evaluating the deviation of an individual relative to others. The Procrustes distance was used to assess morphological divergence between group mean shapes. Statistical significance was assessed by 10,000 permutation tests. To examine the effect of habitat type on morphology, CVA was performed with specimens grouped into river and lake habitats, and the accuracy of both original and cross-validated classification were computed.

All statistical analyses were carried out in R (v4.4.1) and MorphoJ (v1.08) software. The geomorph R package (v4.0.1) was used for landmark data analysis and visualization, while the vegan (v2.7-1) and pls (v2.8-5) R packages facilitated multivariate statistical testing. GPA, PCA, CVA, mean shape calculation, and TPS deformation grid generation were conducted in MorphoJ (v1.08) [25,35].

### 2.4. Correlation of Morphology with Geographical Distance and Environmental Factors

Mantel test was applied to assess the correlation between morphological distance and geographical distance [36]. The morphological distance matrix was computed as the Euclidean distance between specimens based on GPA-aligned Procrustes coordinates. The geographical distance matrix was calculated from the longitude and latitude coordinates of the sampling sites using the Haversine formula. The correlation was evaluated using the Pearson method, with significance determined by 9999 permutation tests.

Environmental variables included mean annual water temperature, mean annual flow velocity, water depth, and food abundance (Table S2). Data for temperature, flow velocity, and depth were sourced from the National Earth Data Science Center (https://www.geodata.cn, accessed on 1 May 2025). Food abundance was scored on a 0–5 scale according to the Evaluation System for Aquatic Biological Integrity in the Yangtze River Basin (Table S3). Partial least squares regression (PLS) model was implemented to predict phenotypic variation from environmental factors [12]. Geometric morphometric data were first reduced by PCA, and PCs explaining ≥ 95% of cumulative variance were retained as response variables. Environmental factors were standardized and one-hot encoded where necessary, then used in PLS modeling against morphological PCs. Model performance was evaluated through cross-validation, and the key environmental variables were identified based on variable importance in projection (VIP).

## 3. Results

### 3.1. Data Reliability Validation

The data quality control indicated high consistency in operator re-landmarking (Table S4), with an intraclass correlation coefficient (ICC) of 0.9268, exceeding the acceptable threshold of 0.75 [33]. Procrustes ANOVA (Table 1) revealed no significant measurement error (residual MS = 0.000038 < individual MS = 0.000129). Strong agreement between Procrustes sum of squares (0.0935) and tangent space sum of squares (0.0933) further confirmed the reliability of landmark data for geometric morphometric analyses.

### 3.2. Geometric Morphometric Differentiation Among Populations

**Figure 3:**
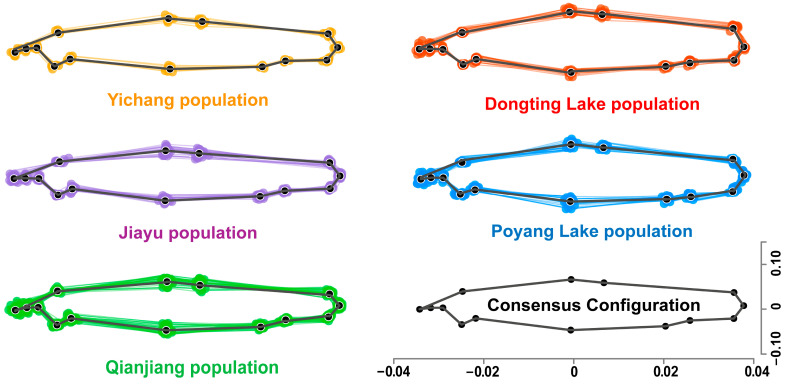
Generalized Procrustes Analysis (GPA) of the *O. elongatus* populations.

**Figure 4:**
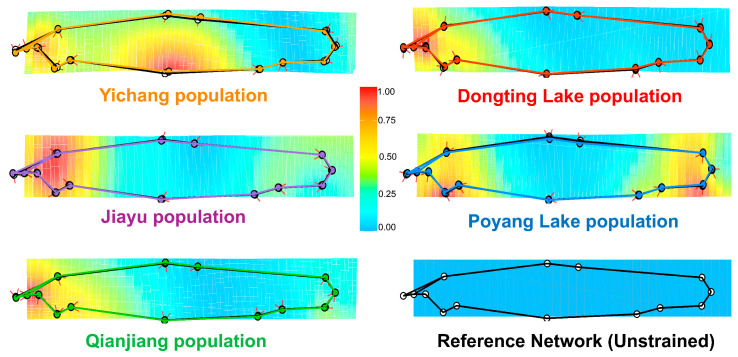
Thin-plate spline analysis (TPS) of the *O. elongatus* populations.

GPA analysis indicated deviations from the consensus configuration in all populations. River populations (Yichang, Jiayu, and Qianjiang) exhibited a more elongated body shape, while lake populations (Dongting and Poyang Lake) showed a relatively deeper body (Figure 3). TPS results further revealed phenotypic variation among populations, with regions of high phenotypic disparity localized in the head (oral position and operculum), the abdomen (origin of pelvic fin) and the tail (caudal peduncle). Specifically, river populations exhibited a smaller head, lower oral position, larger operculum, and narrower caudal peduncle, whereas lake populations were characterized by a more anterior origin of pelvic fin (Figure 4).

**Figure 5:**
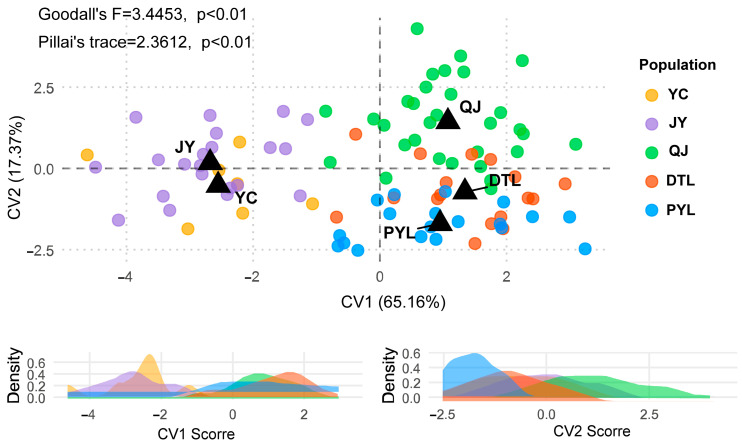
Canonical variate analysis (CVA) of phenotypic variation among *O. elongatus* populations from different habitats. Population abbreviations: YC: Yichang, JY: Jiayu, QJ: Qianjiang, DTL: Dongting Lake, PYL: Poyang Lake.

The first two canonical variates collectively accounted for 82.53% of the phenotypic variation (CV1: 65.16%; CV2: 17.37%). Populations exhibited clear phenotypic segregation by different habitat types. The mainstem populations (Yichang and Jiayu) clustered toward the negative axis of CV1, and lake populations (Dongting and Poyang Lake) occupied the positive axis of CV1, while the tributary population (Qianjiang) aligned toward the positive axis of CV2 (Figure 5).

**Figure 6:**
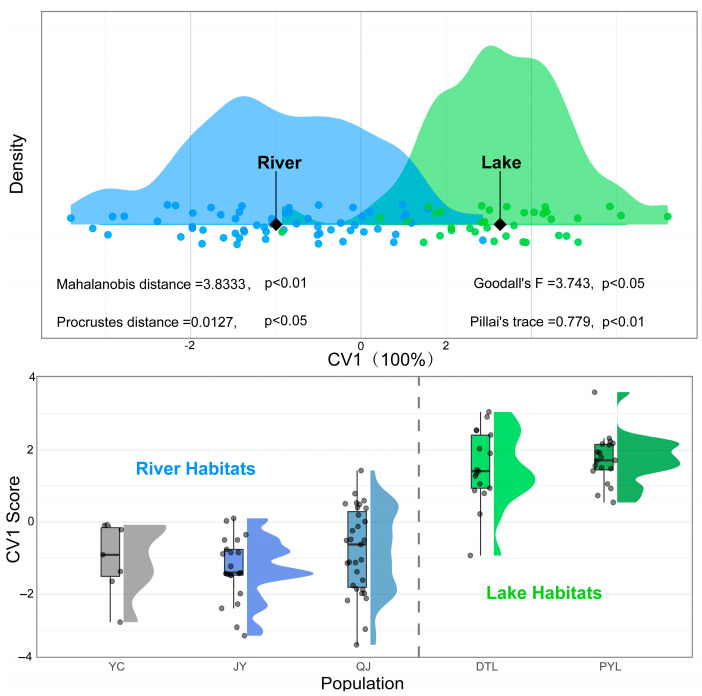
Canonical variate analysis (CVA) of morphometric differentiation in *O. elongatus* between river and lake habitats. Population abbreviations: YC: Yichang, JY: Jiayu, QJ: Qianjiang, DTL: Dongting Lake, PYL: Poyang Lake.

To clarify phenotypic differences between river and lake habitats of *O. elongatus*, populations from Yichang, Jiayu, and Qianjiang were combined into a river group, while those from Dongting and Poyang Lake were combined into a lake group for CVA analysis. The CV1 alone could explain all variations between the river and lake group (eigenvalue = 3.5325), and significant differentiation was detected between the two groups (Pillai’s trace = 0.779, *p* < 0.01; Goodall’s *F* = 3.743, *p* < 0.05) (Figure 6). Both the Mahalanobis distance (3.833, *p* < 0.01) and Procrustes distance (0.013, *p* < 0.05) were significant, further supporting pronounced phenotypic differentiation between river and lake populations.

Discriminant analysis based on river and lake habitat type showed high classification accuracy, with original and cross-validated accuracy rates of 95.8% and 91.6% (Table 2), respectively, indicating a strong association between phenotypic variation and river versus lake habitat types.

### 3.3. Influence of Geographic and Environmental Variables on Morphological Traits

**Figure 7:**
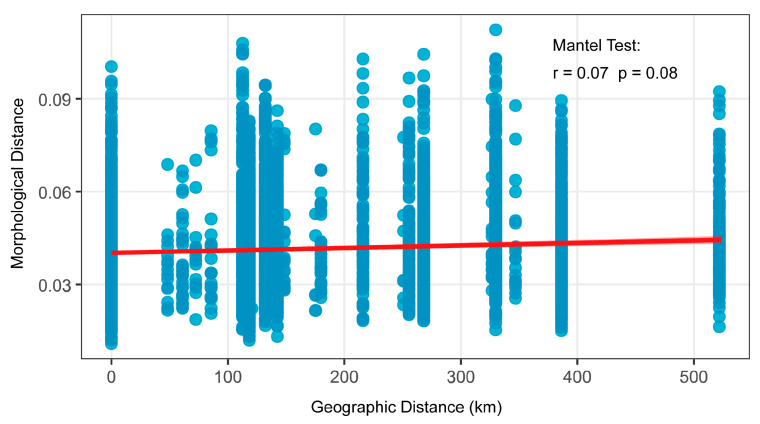
Correlation between morphological distance and geographical distance.

The Mantel test revealed no significant correlation between morphological distance and geographical distance in populations of *O. elongatus* (r = 0.07, *p* = 0.08) (Figure 7).

**Figure 8:**
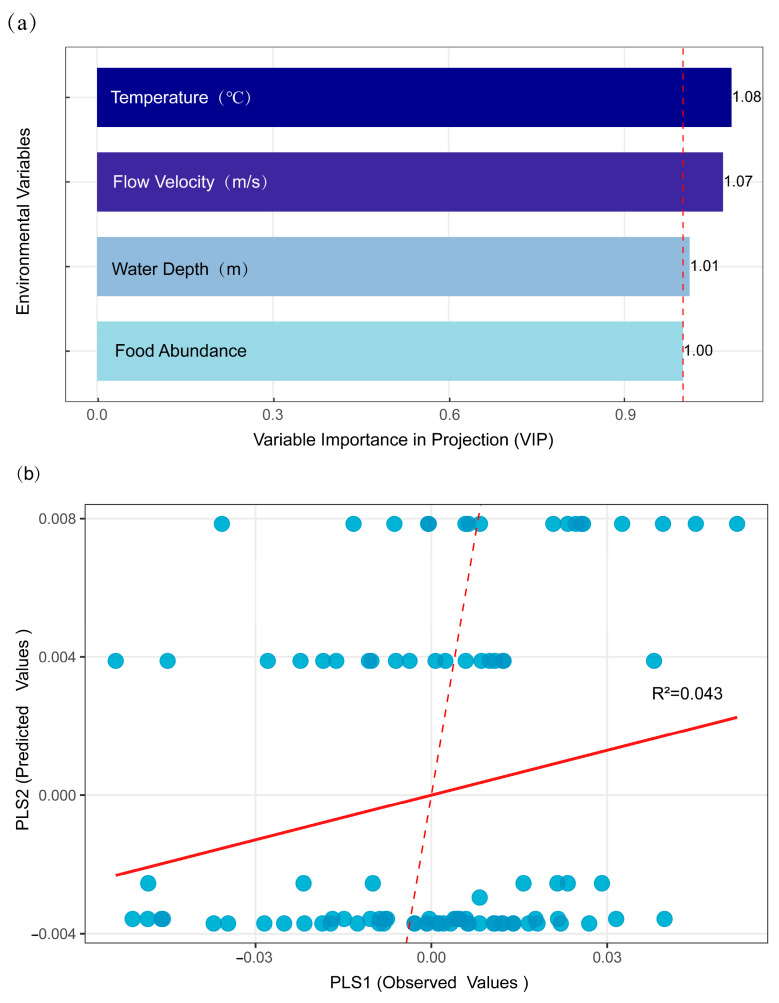
Partial least squares (PLS) regression analysis of phenotypic variation and environmental factors. (**a**) Variable importance in projection (VIP) scores of environmental factors. Factors with VIP ≥ 1 (dashed line) were considered influential. (**b**) Scatter plot of predicted vs. observed morphological values. The solid red line indicated the fitted regression, and the dashed red line represented the line of ideal prediction.

VIP analysis indicated that temperature, flow velocity, water depth, and food abundance all exerted significant influences on phenotypic variation (VIP ≥ 1). However, all VIP values were only marginally above the significance threshold, with limited individual contributions (Figure 8a). The comparison between observed and predicted values demonstrated a weak overall model explanation (R^2^ = 0.043), indicating that environmental factors accounted for only 4.3% of the total phenotypic variation (Figure 8b).

## 4. Discussion

Phenotypic variation in fish often reflects functional and ecological adaptations. This study revealed significant differentiation across multiple morphological regions among populations of *O. elongatus* in the middle Yangtze River, with the highest divergence observed in the head, the abdomen, and the caudal peduncle. As a key structure for feeding and sensory functions, the variation in head was associated with dietary divergence and foraging strategies. Differential food resources could induce corresponding adaptive head morphologies; for instance, Andersson [37] demonstrated in controlled experiments that *Salvelinus alpinus* feeding on zooplankton developed smaller heads, whereas those consuming benthic chironomid larvae exhibited larger heads. Hernandez et al. [12] suggested that fish in river environments developed larger operculum to cope with oxygen availability. Abdominal morphology, particularly the position of the pelvic fins, was linked to swimming stability and habitat complexity. Andersson et al. [38] reported that fish inhabiting structurally complex environments tended to have more anteriorly positioned pelvic fins, which facilitated rapid stopping, turning and hovering, thereby enhancing maneuverability. Caudal peduncle morphology was closely tied to swimming and escape behavior, and varied with flow velocity. Studies on species such as *Culter alburnus* and *Salmo salar* had identified the caudal peduncle as a region of high variability [39,40].

In this study, river populations (Yichang, Jiayu, and Qianjiang) exhibited a more elongated body shape, whereas lake populations (Dongting and Poyang Lake) had a deeper body and more anterior origin of pelvic fins. The river sections in the middle Yangtze were characterized largely by unidirectional flow and have become structurally simplified due to channel dredging and dam construction. In contrast, Dongting and Poyang Lakes exhibited complex hydrology and diverse habitats. These findings aligned with the documented patterns wherein fish in simple habitats tended to be more elongated, while those in complex environments displayed deeper bodies and anteriorly shifted pelvic fins [41]. Our stomach content analysis indicated that the Jiayu and Qianjiang populations primarily consumed zooplankton, whereas the Poyang Lake group incorporated fish into their diet (unpublished data), suggesting a trophic divergence between river and lake populations. This may partly explain the observed differences in head and oral morphology. Furthermore, the larger operculum and narrower caudal peduncle in river populations were consistent with previously reported adaptations to flowing water. Therefore, the phenotypic divergence observed in the head, abdominal, and caudal regions of *O. elongatus* reflected functional adaptations related to locomotion and feeding, illustrating phenotypic plasticity in response to local habitat conditions and resource availability.

The selective role of divergent habitats in driving phenotypic differentiation has been documented across various fish species. For instance, Li et al. [42] identified lake, marine, and river ecotypes of *Coilia nasus*, while Wang et al. [43] reported distinct lake and river ecotypes in *Schizopygopsis stoliczkai*. Similarly, studies by Langerhans et al. [8] and Hernandez et al. [12] on neotropical fishes (*Bryconops caudomaculatus* and *Biotodoma wavrini*) and *Caquetaia kraussii*, revealed phenotypic differentiation between channel–lake and river–swamp habitats, respectively. These findings suggested that such ecological divergence might be widespread among fishes. Our study revealed a consistent pattern in the *O. elongatus* populations, in that phenotypic structures were significantly influenced by habitat type, allowing classification into mainstem, tributary, and lake groups. The phenotypic variation followed a gradient along habitat types, while minimal divergence between the mainstem and tributary populations was shown, so we merged the mainstem and the tributary samples into a river group and the two lake samples into a lake group to enhance clarity in distinguishing river and lake phenotypic differentiation. Discriminant analysis based on river and lake habitat types showed that the CV1 effectively distinguished river and lake groups, with the accuracy of both original and cross-validation classification exceeding 90%. This strongly supported the finding that the phenotypic differentiation between river and lake populations of *O. elongatus* in the middle Yangtze River were not random but reflected significant adaptive divergence.

The Mantel test revealed no significant correlation between morphological distance and geographical distance in the *O. elongatus* populations, indicating that geographical distance was not the primary driver of phenotypic differentiation. This finding contrasted with the common isolation by distance pattern in many freshwater fish species. Zhai et al. [24] indicated extensive gene flow among populations of *O. elongatus* in the middle Yangtze River, with no significant genetic differentiation observed. Previous studies had shown that gene flow between populations could constrain genetic differentiation, but it did not necessarily limit phenotypic divergence resulting from phenotypic plasticity [44]. In this study, significant phenotypic differentiation was detected between river and lake populations, suggesting that habitat heterogeneity was a key factor promoting phenotypic divergence. Analysis of environmental variables indicated that temperature, flow velocity, water depth, and food abundance all significantly influenced phenotypic variation, but each factor exhibited limited independent explanatory power. This implied that environmental shaping of morphology likely depended on synergistic effects of multiple factors, which was consistent with the findings of Hetzel and Forsythe [45] in *Semotilus atromaculatus*, where phenotypic plasticity was often regulated by a combination of environmental cues rather than a single dominant factor. The multi-factor synergy might reflect an adaptive strategy to replicated environment, as these factors were often interdependent (e.g., water temperature affects food distribution; flow velocity is correlated with depth) and might collectively shape phenotype through indirect pathways such as behavioral adjustments and energy allocation. Moreover, the overall explanation of environmental factors was low, indicating that some unquantified factors might contribute to the unexplained phenotypic variation.

## 5. Conclusions

Our study revealed significant phenotypic differentiation between river and lake populations of *O. elongatus* in the middle Yangtze River, reflecting local adaptation and phenotypic plasticity in response to distinct hydrological environments. The pattern of phenotypic divergence was closely associated with habitat type. The river populations exhibited a more elongated body shape, whereas lake populations displayed a deeper body, with notable variations in functional regions such as the head, abdomen, and caudal peduncle. These variations suggested that *O. elongatus* could modulate its body structure to cope with different flow regimes and adjust head morphology to exploit varied foraging resources, demonstrating phenotypic plasticity that enabled broad adaptation to diverse habitats. These findings provide important insights into the adaptive evolution of this critically endangered species and offer a scientific basis for conservation strategies. Based on our findings, we propose that the *O. elongatus* population in the middle Yangtze River should be divided into two management units: a riverine unit and a lacustrine unit. For the riverine unit, we recommend maintaining natural hydrological regimes and river connectivity, protecting critical habitats such as spawning sites, and mitigating impacts from dams and embankments. For the lacustrine unit, efforts should emphasize integrated aquatic ecosystem conservation, including water quality management and regulation of water levels, to sustain its adapted habitats.

## Figures and Tables

**Table 1:** Procrustes-based analysis of variance in geometric morphometrics. Values include sum of squares (SS), mean square (MS), degrees of freedom (df), and *F*-statistic.

Centroid Size:							
**Effect**	**SS**	**MS**	**df**	***F***	***p* (param.)**		
Individual	866,562.758	216,640.690	4	27.43	<0.01		
Residual	710,864.676	7898.496	90				
**Shape, Procrustes ANOVA:**							
**Effect**	**SS**	**MS**	**df**	***F***	***p* (param.)**	**Pillai tr.**	***p* (param.)**
Individual	0.012400	0.000129	96	3.45	<0.01	2.36	<0.01
Residual	0.080980	0.000038	2160				

**Table 2:** Classification accuracy for *O. elongatus* from river versus lake habitats.

Classification Method	Group	Correct Size	Misclassified Size	Accuracy	Overall Accuracy
Original Classification	River	57	2	96.6%	95.8%
	Lake	34	2	94.4%	
Cross-Validation	River	54	5	91.5%	91.6%
	Lake	33	3	91.7%	

## Data Availability

Data are contained within the article and Supplementary Materials.

## Supplementary Materials

The following supporting information can be downloaded at: https://www.mdpi.com/article/10.3390/ani15192870/s1, Table S1: Sampling information of *O. elongatus*; Table S2: Geographic coordinates and environmental factors of sampling sites for *O. elongatus* populations in the middle Yangtze River; Table S3: Scoring criteria for the food abundance based on biological indicators; Table S4: Operator consistency analysis summary.
