# Supplementary material for: Revealing Phenotypic Differentiation in Ochetobius elongatus from the Middle Yangtze River Through Geometric Morphometrics
Source: Animals (Basel). 2025 Sep 30;15(19):2870. doi: 10.3390/ani15192870 (PMC12524143; doi:10.3390/ani15192870)
Supplement: Supplementary file 1 [file animals-15-02870-s001.zip › animals-3875173-supplementary.pdf]

Table S1. Sampling information of *O. elongatus.*

Sampling site /Code	River system	Sample size	Body length (cm)	
			Range	Mean±SD
Yichang /YC	Mainstem	7	32.9~45.9	40.6±47.7
Jiayu /JY	Mainstem	21	16.0~21.1	18.4±13.3
Qianjiang /QJ	Tributary	31	15.5~22.2	17.8±97.5
Dongting Lake /DTL	River-connected Lake	17	32.5~43.1	36.2±29.7
Poyang Lake /PYL	River-connected Lake	19	20.9~50.0	30.7±88.0

Table S2. Geographic coordinates and environmental factors of sampling sites for *O. elongatus* populations in the middle Yangtze River.

Population	Longitude (°E)	Latitude (°N)	Water Temperature (°C)	Flow Velocity (m/s)	Water Depth (m)	Food Abundance
Yichang	111.341	30.639	18.5±0.4	1.4±0.2	12.5±0.3	3
Jiayu	113.890	29.992	20.0±0.3	0.8±0.1	9.3±0.5	3
Qinajiang	112.818	30.515	20.5±0.3	0.5±0.3	6.4±0.7	3
Dongting Lake	113.003	29.335	19.5±0.8	0.2±0.2	6.8±1.2	5
Poyang Lake	116.356	28.900	21.0±1.0	0.3±0.1	8.2±1.8	5

Notes: Water temperature, flow velocity, and water depth referred to mean annual water temperature, mean annual flow velocity, and water depth, respectively. Food Abundance was a comprehensive reflection of the biomass and biodiversity. A higher value indicated greater food abundance. The detailed criteria for these values were provided in Table S3.

Table S3. Scoring criteria for the food abundance based on biological indicators

**1. Phytoplankton Biomass (Mean abundance: 10^6 cells/L)**						
Score	0	1	2	3	4	5
Current value	≥100	[50, 100)	[10, 50)	[5, 10)	[2, 5)	<2
**2. Zooplankton Biomass (Ratio of Current to Reference Value)**						
Score	0	1	2	3	4	5
Ratio	≤20%	(20%,35%]	(35%,50%]	(50%,65%]	(65%,80%]	>80%
**3. Mollusk Species Richness (Ratio of Current to Reference Value)**						
Score	0	1	2	3	4	5
Ratio	≤20%	(20%,30%]	(30%,40%]	(40%,60%]	(60%,80%]	>80%
**4. Benthic Diversity (Shannon-Wiener Index H). Deviation = |(Current H - Reference H)| / Reference H × 100%**						
Score	0	1	2	3	4	5
Deviation	≥50%	(50%, 60%]	(60%, 70%]	(70%, 80%]	(80%, 90%]	>90%

Notes: Reference values were established based on pristine or minimally disturbed conditions. The scores (0-5) represented the integrated assessment of food abundance.

Table S4. Operator Consistency Analysis Summary.

**Analysis Indicator**	**Value**	**Explanation**
Operator Effect-*F* value	204.3424	*F*-statistic from ANOVA
Operator Effect-Degrees of Freedom	(1, 19)	Model degrees of freedom
Operator Effect-*p* value	0.0010	Significance of operator effect
ICC (2,1)	0.9268	Inter-rater reliability
Between-Individual Variance	0.0197	Biological variation between samples
Between-Operator Variance	0.0014	Systematic bias between operators
Measurement Error	0.0001	Random variation in measurement
